# DNA Metabarcoding for the Characterization of Terrestrial Microbiota—Pitfalls and Solutions

**DOI:** 10.3390/microorganisms9020361

**Published:** 2021-02-12

**Authors:** Davide Francioli, Guillaume Lentendu, Simon Lewin, Steffen Kolb

**Affiliations:** 1 Microbial Biogeochemistry, Research Area Landscape Functioning, Leibniz Centre for Agricultural Landscape Research (ZALF), Eberswalder Str. 84, 15374 Müncheberg, Germany; Simon.Lewin@zalf.de (S.L.); steffen.kolb@zalf.de (S.K.); 2 Laboratory of Soil Biodiversity, University of Neuchâtel, Rue Emile-Argand 11, 2000 Neuchâtel, Switzerland; guillaume.lentendu@unine.ch

**Keywords:** DNA metabarcoding workflow, high-throughput sequencing, terrestrial ecosystem, bacteria, archaea, fungi, protists, soil and plant-associated microorganisms

## Abstract

Soil-borne microbes are major ecological players in terrestrial environments since they cycle organic matter, channel nutrients across trophic levels and influence plant growth and health. Therefore, the identification, taxonomic characterization and determination of the ecological role of members of soil microbial communities have become major topics of interest. The development and continuous improvement of high-throughput sequencing platforms have further stimulated the study of complex microbiota in soils and plants. The most frequently used approach to study microbiota composition, diversity and dynamics is polymerase chain reaction (PCR), amplifying specific taxonomically informative gene markers with the subsequent sequencing of the amplicons. This methodological approach is called DNA metabarcoding. Over the last decade, DNA metabarcoding has rapidly emerged as a powerful and cost-effective method for the description of microbiota in environmental samples. However, this approach involves several processing steps, each of which might introduce significant biases that can considerably compromise the reliability of the metabarcoding output. The aim of this review is to provide state-of-the-art background knowledge needed to make appropriate decisions at each step of a DNA metabarcoding workflow, highlighting crucial steps that, if considered, ensures an accurate and standardized characterization of microbiota in environmental studies.

## 1. Introduction

Soil microorganisms have been recognized as an integral part of terrestrial ecosystems because they play a central role in nutrient transformation and in plant community productivity, composition and diversity [1]. However, our knowledge of soil microbiota is limited by the huge microbial diversity that characterizes terrestrial ecosystems and by the complexity of soil–plant–microbe interactions [2]. Indeed, soil has often been dubbed a “black box” because of the high abundance of soil microbial populations (10^8^–10^11^ cells per gram) and the methodological challenges to characterize them [3,4]. Currently, this black box is beginning to be pried open, largely due to advances in molecular tools that have paved the way forward for soil microbial ecologists to unravel the composition and function of the soil microbiota in terrestrial ecosystems [5]. Novel molecular approaches, which employ polymerase chain reaction (PCR) and high-throughput sequencing (HTS), have revolutionized the way to study the soil microbiota. Application of these methods has demonstrated that a large fraction of terrestrial microbes can be detected solely using molecular approaches, thus discouraging the need for laboratory isolation and culturing of specimens. Furthermore, with the decrease of sequencing price and high-throughput samples analysis by various bioinformatics tools, the use of massively parallel sequencing (MPS) in soil microbial ecology has become a standard approach. 

Prokaryotes (Archaea and Bacteria) and fungi are the most studied microbes in soils and plants. The “other” microbes in soils are grouped under the term protists [6], and despite their relative lower abundance compared to their prokaryotic and fungal counterparts, they carry significant functional roles at all trophic levels [7]. The characterization of the soil microbial community is commonly carried out via PCR amplification of taxonomic marker genes (called “DNA barcodes”). These markers are typically 100 to 600 bp long, and they need to be sufficiently variable to provide deep taxonomic resolution and are simultaneously flanked by conserved regions to cover a broad range of taxa. The combination of HTS with barcoding has been named “metabarcoding” [8]. The relative short length of these markers does not always allow a resolution to species level, so alternative approaches like single-cell metagenomics or isolation via cultivation are needed to fully discriminate microbial species. Despite this limitation, DNA metabarcoding has rapidly emerged as a powerful, repeatable and cost-effective method for characterizing microbial communities in small and large-scale studies. This comprehensive approach has enabled soil microbiologists to explore important ecological aspects related to soil–plant–microbe systems, such as the identification of microbial taxa that are (i) dominant or low in abundance across different terrestrial ecosystems; (ii) involved in specific processes (e.g., litter decomposition, nitrogen cycling, degradation of toxic compounds and many more); (iii) more sensitive to abiotic and biotic factors. DNA metabarcoding further allows assessing soil microbial biodiversity (also in terms of phylogenetic relatedness), and to compare soil communities subjected to experimental conditions or geographical distance. It is also a cost-effective method for biomonitoring as DNA metabarcoding is more frequently used for monitoring agricultural practices, restoration efforts or forensics [9,10,11,12]. Presently, it represents the most used molecular approach to characterize microbiota in environmental samples. 

**Figure 1:**
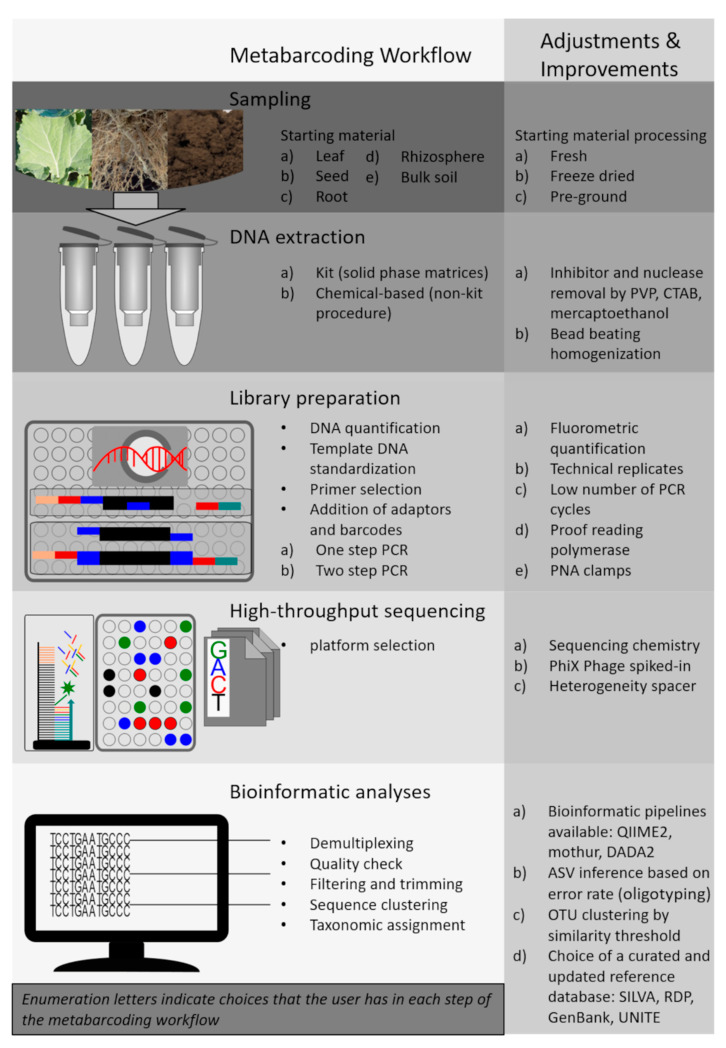
DNA metabarcoding workflow with suggested adjustments and improvements.

In this review, we focus on all the steps in the identification of soil and plant-associated microbes using DNA metabarcoding (Figure 1). This approach consists of multiple laboratory procedures and requires bioinformatics and computational statistics. Therefore, sufficient technical knowledge and informed choice at each step are essential for successful microbial detection and taxonomic identification [13]. In addition, the use of DNA metabarcoding for microbial identification has some important limitations, including the variable number of copies of the selected gene markers in microbial genomes, the low taxonomic resolution at the species level for some microbial groups and biases in the taxonomic annotation of sequences depending on the variable region chosen for the analysis [14,15]. Hence, the choice of a proper modus operandi for all the steps in metabarcoding workflows is crucially important. Inappropriate methods in microbiota studies may generate insufficient and fallacious biological inferences [16,17]. Indeed, significant biases can occur from the cumulative effect of both systematic and random errors throughout the whole workflow, including sampling, DNA extraction, amplicon library preparation, sequencing and bioinformatics [18,19].

Based on literature review and experience, we provide a comprehensive overview of the positive and negative aspects related to each step of the metabarcoding workflow for microbiota studies on samples associated with terrestrial ecosystems (Figure 1). Since sampling procedures for soil- and plant-associated microbiome were already covered in other reviews [18,20,21], we here concentrate mainly on the molecular aspects of the metabarcoding workflow. Therefore, in the next sections, we first discuss practical sample handling procedures and molecular approaches fundamental in the preparation of the sequencing library. This will provide guidance on important methodological issues that might be overlooked. Second, we describe useful software tools that are typically employed in the bioinformatics data processing and in the taxonomic characterization of the detected microbial taxa. Finally, we discuss potential future applications of next-generation sequencing (NGS) platforms and technologies in unraveling the relationships between microbial biodiversity and ecosystem functions. 

## 2. DNA Extraction Procedure

Extraction of the genetic material from environmental samples is the first step in the metabarcoding workflow. Total genomic DNA extraction represents a crucial stage in which the potential biases have to be minimized using appropriate laboratory protocols. The analytical success of molecular techniques is significantly affected by a successful DNA extraction, which involves the effective sample homogenization and disruption of cells, denaturation of proteins and nucleoprotein complexes, inactivation of nucleases, removal of humic acids and other PCR inhibitors and recovery of the DNA. Presently, these steps are performed using commercial kits that employ both chemicals and solid-phase matrices. Such DNA extraction kits are simple to use and rapid, and most of them do not include harmful solutions. However, chemical-based DNA extraction protocols that do not involve the use of commercial kits, such as phenol-chloroform-based extraction method, are still in use [22]. Such DNA extraction procedures are usually cheaper per extraction compared to commercial kits, in addition to their good quality and quantity of the extracted DNA. Moreover, the different steps and solutions of such a procedure can be optimized to the sample material. However, solution-based DNA extraction protocols can be quite laborious, since (i) all the steps are manual, (ii) they often need fresh-made solutions and (iii) they use toxic chemicals.

For the isolation of total genomic DNA from terrestrial environments (soil and plant material), many commercial kits and protocols for soil, seeds and plant tissue are available (Table 1). However, the choice of the suitable DNA extraction procedure can be more complicated when dealing with multiple sample types such as bulk soil, rhizosphere, stem and leaf. In the case that the experimental aim is the comparison of microbiota across compartments, then the DNA associated with such compartments must be extracted with the same method. This is necessary to avoid protocol-specific biases when comparing, for example, rhizospheric soil to root or either of those to the leaf or stem tissue. However, each compartment can be extracted with the method that works best for it when the comparison across compartments is not the aim. This will provide a better snapshot of the community associated with each compartment, but with the loss of the capability to compare between them. Based on our experience, the soil DNA extraction kits listed in Table 1 can be employed for the extraction of genomic material from different types of samples (soil, sediments and plant material) with satisfactory results in terms of quantity and quality of the DNA.

Another important aspect to consider concerning the DNA extraction procedure is that Gram-positive and -negative Bacteria, Archaea, fungi and protists are differentially sensitive to cell disruption. Thus, sample homogenization and disruption of cells can represent a major cause of bias in the microbiota composition. Thereby, bead-beating in combination with chemical lysis agents was shown to be most efficient for soil and plant material [23]. Thus, the downstream analyses will not be confounded to less or highly resistant microorganisms. Furthermore, when there is the need to process a large number of samples, bead beating-based kits can represent a much better choice than tedious and time-consuming “home-made” protocols (e.g., phenol-chloroform-based methods), although the kits tend to be more costly. It is worth mentioning that the bead beating procedure requires a dedicated bead beater homogenizer, which can be prohibitive due to its cost (from a few thousand up to 10,000 dollars depending on the homogenizer features). 

Additionally, other extraction methodologies can be employed when the objective is to extract not the soil total genomic DNA (also known as environmental DNA or eDNA [24]) but specific fractions of it. For instance, to collect the extracellular DNA fraction, which can be released from dead prokaryotic and eukaryotic cells and can be protected against nuclease degradation by its adsorption on soil colloids and sand particles, protocols that avoid the lysis of the cells by using only low centrifugation speeds and mild chemical concentrations are generally used [25,26]. Another approach, named “indirect DNA extraction”, is employed when the aim is to individually collect different microbial DNA fractions. This method involves the initial separation of prokaryotic and eukaryotic cells from the soil matrix by density gradient centrifugation prior to their lysis [27,28]. Such isolated cell communities could then be further sorted at the single-cell level using flow cytometry or microfluidic devices before DNA extraction and subsequent metabarcoding [29,30].

Therefore, the choice of a particular DNA extraction protocol depends on the type and number of samples, study purpose, equipment availability and financial constraints. Finally, the extracted DNA can be stored at −20 °C or −80 °C for further processing. It is also worth noting that RNA could be extracted in parallel to DNA using dedicated kits, but this aspect was covered elsewhere [31,32,33] and we will not go into detail here.

**Table 1:** Commonly used DNA extraction kits and methods for soil and plant-associated microbiota.

Kit Manufacturer or Method	Sample Type	Homogenization and Cell Lysis	DNA Purification and Concentration	Relative Cost Per Sample [Low ($) to High ($$$)]
*DNeasy PowerSoil* Qiagen, USA	Soil, compost, manure, plant material	Bead beating + chemical lysis	Silica membrane binding	$$$
*FastDNA Kit for Soil* MP Biomedicals, USA	Soil, compost, manure	Bead beating + chemical lysis	Silica membrane binding	$$$
*Plant DNeas*y *Mini kit* Qiagen, USA	Plant and fungal tissue.	Mortar/pestel or TissueLyzer + chemical lysis	Silica membrane binding	$$
*Quick-DNA Fecal/Soil Microbe Miniprep Kit* Zymo Research, Germany	Soil, biofilm, animal and human samples	Bead beating + chemical lysis	Silica membrane binding	$$
Phenol-chloroform-isoamyl alcohol-Extraction [22]	Soil	Bead beating + CTAB ^a^	PEG ^b^ 6000 + ethanol precipitation	$
Phenol-chloroform-isoamyl alcohol-Extraction modified [31]	Soil	Bead beating + CTAB ^a^ + PVP ^c^	PEG ^b^ 6000 + ethanol precipitation	$
Phenol-chloroform-isoamyl alcohol-Extraction modified [32]	Soil	Bead beating + CTAB ^a^ + PVPP ^d^	Isopropanol precipitation	$$
Sodiumphosphate extraction [34]	Sediments	Bead beating + Sodiumphosphate buffer + PVP ^c^	Silica membrane binding + GuaHCL ^e^ precipitation	$$

^a^ hexadecyltrimethylammonium bromide; ^b^ polyethylene glycol; ^c^ polyvinylpyrrolidone; ^d^ polyvinylpolypyrrolidone; ^e^ guanidium-hydrochlorid.

## 3. Amplicon Library Preparation

### 3.1. DNA Quality and Quantity

The next step in the metabarcoding workflow is the preparation of the sequencing library. In this stage, several key points deserve careful consideration regardless of the sequencing platform that will be employed once the amplicon library is complete. First, the DNA template that will be used for the subsequent PCRs should be checked for its quality and quantity. The easiest way to assess DNA quality is by a spectrophotometer. Nucleic acids (DNA and RNA) absorb maximally at a wavelength of 260 nm. Protein absorbs best at 280 nm and organic compounds and chaotropic salts at 230 nm. In general, the A260/A280 ratio is used as an indicator of DNA purity, and its value should range between 1.8 and 2.0. The A260/A230 ratio is also a metric for DNA quality, and it is best if it is greater than 1.5. If these ratios are appreciably lower in either case, it may indicate the presence of protein, phenol or other contaminants that may be introduced by extraction procedures and can act as PCR inhibitors. To overcome PCR inhibition due to a low purity of the extracted DNA, preliminary PCR tests using a serial dilution of template DNA, additional purification procedures (commercial kit or manual ethanol, isopropanol, polyethylene glycol precipitation) and/or addition of PCR-enhancing or -stabilizing agents (dimethyl sulfoxide, betaine, bovine serum albumin) can be performed. As alternatives, PVP (polyvinylpyrrolidone) and 2-mercaptoethanol can be added during the cell lysis step in the DNA extraction procedure to remove negatively charged polysaccharides and polyphenols. 

For accurate quantification of the extracted DNA, the use of fluorimetric determination is recommended, which utilizes fluorescent dyes that bind to double-strand DNA. This quantification method is more sensitive than using a spectrophotometer, especially when samples with low, e.g., nanomolar, DNA concentration are measured. After DNA quantification, it is recommended to standardize DNA concentrations prior to PCR, because DNA concentration might be highly variable among samples. The importance of this latter step is to have approximately the same amount of template DNA for the subsequent PCR amplification. Typically, 10–20 ng of template DNA is sufficient for amplification of ribosomal marker genes (see below for further details), but higher amounts might be required if rare gene markers are targeted [35].

### 3.2. Amplification of a Target Marker Gene

The success of DNA metabarcoding mainly depends on the selection of the appropriate DNA marker gene, which requires careful consideration. Ideally, such gene markers should have sufficiently conserved flanking primer-binding sites to minimize taxonomic bias during PCR amplification, while the intervening sequence is sufficiently variable for taxonomic identification [36]. In silico PCR is thus a critical step in the development of a primer in order to control for appropriate coverage of the target group (i.e., taxonomic coverage and breath), the efficient exclusion of outgroups (i.e., taxonomic specificity) and the ability to discriminate taxa based on nucleotide variability of the amplified marker (i.e., taxonomic resolution). Integrated tools, such as TestPrime [37], are available to perform in silico PCR directly on a specific database (e.g., SILVA rDNA database). More generic tools that search for primers can be used on any set of reference sequences and allow for the computation of standard coverage and specificity indices, like ecoPCR or cutadapt [38,39].

Moreover, amplicon length is a critical aspect, as longer sequences will substantially increase annotation accuracy and phylogenetic resolution [40]. Amplicon libraries created for being sequenced using Illumina paired-end technology will produce amplicon sizes up to 2 × 300 bp. For longer amplicons, third-generation NGS technology, such as those of Pacific Biosciences [41] and Oxford Nanopore Technologies [42], can be employed. The major advantage of third-generation NGS technology over broadly established technologies is the capability to produce ultra-long reads spanning genomic fragments measured in tens of thousands of bases [43]. At present, the benefits of the third-generation sequencing come at cost of sequencing accuracy [44]. However, Illumina technology is, so far, the most accurate technology that has been used in nearly all metabarcoding studies. It provides reads of 100 to 500 bp, which in most cases is sufficient for the analysis of typical gene markers, such as the informative regions of 16S/18S rRNA gene of prokaryotes/eukaryotes or the ITS region of fungi. Hence, we will focus only on amplicons library preparation conceived for Illumina sequencing in the next sections. 

#### 3.2.1. Identification of Prokaryotes from Environmental Samples

Characterization of prokaryotic communities (Bacteria and Archaea) in environmental samples targeting regions of the 16S rRNA gene has been widely employed, unless primers have been designed to detect individual species and/or genera. 16S rRNA gene primer pairs usually target a single stretch of the hypervariable regions of the ~1500 bp prokaryote 16S rRNA gene [45,46]. Thus, the choice of the hypervariable region (V-region) targeted and the corresponding primer set should be done meticulously in order to provide coverage and accurate representation of the prokaryotic profiles in microbiota analyses [47,48]. In this line, using suboptimal primer pairs can lead to under-representation of certain or selection against single taxa, which can lead to incorrect results and conclusions [49]. The various evaluated primer sets commonly employed to identify bacteria are listed in Table 2. 

Two of the most used sets of primers for soil samples are 515fB [50] and 806rB [51]. This primer pair, which was designed for use with the Illumina platform [60], is recommended for the identification of Bacteria and Archaea from soil samples by the international scientific consortium Earth Microbiome Project (EMP) [61]. However, a recent study on the performance of different Archaea-specific primers reported that the 515fB/806rB primer set performed worst for analysis of Archaea by producing only 2.1% of Archaea reads (on average) and covering only the phyla Euryarchaeota and Thaumarchaeota [62]. This suggests that the diversity of Archaea can been largely underestimated when utilizing the primers 515fB and 806rB, while the primer sets SSU1ArF/SSU520R and 340f/806rB yielded a higher sequencing coverage of the archaeal diversity using Illumina platform [62]. A list of specific primer sets to identify Archaea from soil samples is reported in Table 3.

Several other primer sets have been tested and proposed as suitable candidates for the characterization of Bacteria diversity of soil samples (Table 2). For instance, a recent study [46] reported that the primer pair 341f/B805r [37], which targets the V3 to V4 region, outperformed the other three primer sets in terms of operational taxonomic unit (OTU) numbers, phylogenetic richness and Shannon diversity. The 341f/B805r primers are also recommended in the official protocol for the amplification of 16S rRNA genes released by Illumina [68]. 

The choice of prokaryotic primer pairs becomes more difficult when amplifying regions of the 16S rRNA gene from plant-associated samples. In this type of sample, it is crucial to reduce the amplification of non-target DNA-sequences, such as those co-extracted from plastid (mostly chloroplast) and mitochondria. Hence, the homology between bacterial 16S rRNA gene, mitochondrial and chloroplast 16S rRNA genes complicates the selection of the appropriate primers to study plant–bacteria interactions [48]. The preferred method to reduce the impact of these contaminant sequences is the use of specific mismatching primers, which amplify bacterial 16S rRNA genes while discriminating against chloroplast 16S rRNA genes. The chloroplast mismatch primer 799f [53] has been widely used in combination with the reverse primer 1193r [55] to characterize the bacterial community of plant samples, especially of roots. This primer combination has also revealed the lowest co-amplification levels of chloroplast and mitochondrial 16S rRNA gene reads among the other three bacterial primers tested [46]. It generates ~380 bp amplicons from the hypervariable region V5 to V7 of the bacterial 16S rRNA gene. Mitochondrial 16S rRNA gene amplicons with length of 800 bp are also produced, but they can be easily removed via agarose gel purification. For stem and leaf material, the primer set 799f/1115r [53] can be selected, as recommended in previous works [69,70]. These chloroplast 16S rRNA gene-discriminating primers are commonly utilized for the identification of phyllosphere associated Bacteria [71,72,73] because these primers do not amplify host-plant nor cyanobacterial DNA; cyanobacteria are known to be rare in the phyllosphere [74,75]. 

Alternative techniques, such as the use of peptide-nucleic acid (PNA) PCR-clamps [45] can be employed to reduce the co-amplification of non-target DNA sequences. PNA clamps are synthetic oligomers that bind tightly and specifically to a unique signature in the contaminant sequence and physically block its amplification [76,77]. In brief, they are designed to suppress plant host plastid and mitochondrial 16S rRNA gene contamination in the PCR reaction. For instance, the widely used primer set 515fB/806rB showed a high affinity for chloroplast 16S rRNA gene (up to 97% of the total number of reads) when used to characterize the plant-associated Bacteria from leaves and roots [78]. However, very low chloroplast co-amplification levels have been reported when this primer set is used in combination with PNA clamps [79,80,81], although their employment might also lead to the exclusion of certain microbial taxa [82]. It is worth mentioning that the efficacy of these approaches in reducing host-organelle 16S rRNA gene amplification significantly varies across plant species [83].

#### 3.2.2. Identification of Fungi from Environmental Samples

The common marker DNA sequence used to identify fungi from soil and plant material is the internal transcribed spacer (ITS) region, which has an average length of 500 and 600 base pairs (bp) [84,85]. The ITS region includes the ITS1 and ITS2 sublocus, separated by the 5.8S rRNA gene, and it is situated between the 18S (SSU) and 28S (LSU) rRNA genes in the eukaryotic rRNA cistron [86]. The entire ITS region was described as the genetic marker with the highest probability of successful identification for a very broad range of fungi [87]. Further studies have supported the use of the ITS region as a suitable universal fungal barcode [88,89]. Consequently, most of the environmental and ecological research studies have used and are using the ITS region in combination with NGS for the identification of fungal taxa in environmental samples. Thus, large numbers of ITS sequences have been collected from terrestrial environments that are available in different reference databases, such as UNITE and GenBank (see below in Section 4.2 for more details), making the ITS region the most ubiqutous gene marker for taxonomic characterization of fungal biodiversity. 

However, with the rapid establishment of Illumina technology as the most popular sequencing platform, only short fragments can be sequenced, which constrains the choice to one of the subloci that compose the ITS region, ITS1 or ITS2 (Table 4). Therefore, the primer set selection for the characterization of fungal diversity has created a crucial and critical issue. There is some controversy on the selection of ITS markers for metabarcoding, and yet there is no consensus about which ITS sublocus is the best. Comparisons between ITS1 and ITS2 for fungal profiles have been assessed in many studies, which yielded contrasting conclusions. For example, ITS1 was thought to be more variable and hence should allow for better distinction among fungal species than ITS2 [90,91]. However, the opposite has been shown [92,93]. Nonetheless, both of these ITS regions have meaningful drawbacks and limitations in assessing fungal diversity, such as a taxonomic bias relative to the length of the amplified region, unsuitability for phylogenetic studies, co-amplification of plant DNA and exclusion of specific fungal taxonomic groups [94]. More and detailed information on the differences between the primer sets targeting the ITS1 and ITS2 regions can be found elsewhere (i.e., [95,96,97,98]). 

Although the ITS region has been described, and frequently utilized, as the universal barcode for fungi [87], it has consistently demonstrated poor resolution for the arbuscular mycorrhizal fungi (AMF; phylum Glomeromycota) compared with the 18S rRNA gene (SSU markers) [104]. In Glomeromycota, species are multinucleate with extreme intraspecies divergence in nuclear ribosomal sequences, which creates additional challenges for the use of ITS for species discrimination [105]. Specifically, primer sets targeting the ITS1 sublocus have limited coverage for AMF [106], whereas recent research has highlighted that ITS2 primers can be successfully employed to characterize the most abundant AMF taxa from soil samples [107,108]. However, AMF-specific 18S rRNA gene primers might be able to amplify more families and provide a broader view of the AMF community than fungal ITS2 primers [107]. In this regard, the primer pair AMV4.5NF/AMDGR [109] is widely used to characterize fungal members affiliated with the Glomeromycota using Illumina platforms [110,111,112]. These primers amplify a ~258 bp fragment internal to the 18S rRNA gene. A direct comparison with other AMF-specific primers revealed that the AMV4.5NF/AMDGR outperformed the other tested primer pairs in terms of number of Glomeromycota reads (AMF specificity and coverage) [113,114]. However, these primers tended to preferentially amplify Glomeraceae at the expense of other major families (i.e., Ambisporaceae, Claroideoglomeraceae, Paraglomeraceae) of Glomeromycota [113].

Another disadvantage of the ITS region is its poor resolution for phylogenetic analysis. Diverging levels of genetic variation, due to different rates of evolution, have been observed for the three separate regions (18S rRNA gene, ITS and 28S rRNA gene) that compose the fungal nuclear ribosomal operon. The 18S rRNA gene possesses a low amount of variation among fungal taxa because it evolves slowly compared to the ITS region, which evolves the fastest and exhibits the highest variation among the three rRNA gene regions [115,116]. For phylogenetic analysis at higher taxonomic levels, such as family, order, class and phyla, former studies recommended targeting the 18S regions V1 to V5 with the primer set NS1 and NS4 [99,117]. However, these primers produce sequences of incompatible length for high-throughput sequencing, so new primers targeting the V7-V8 regions of the 18S have been proposed to target fungi in environmental samples when using Illumina sequencing [118]. These primers also have the advantage to cover well the basal fungal groups (i.e., Blastocladiomycota, Chytridiomycota, Entomophthoromycotina, Glomeromycota, Kickxellomycotina, Mucoromycota and Zoopagomycotina) when ITS primers are biased toward Dikarya. Fungal diversity could also be assessed jointly with protists using general eukaryotic primers, particularly the one targeting the V4 18S rRNA gene (see next section, e.g., [119]). The last alternative is to target the 28S rRNA gene with the primer combination LROR and LR3, with the 100 nucleotide (nt) region before the reverse primer being the best discriminant region for fungi [120]. However, this primer pair also amplifies a too-long fragment for Illumina sequencing (~600 nt), so that different strategies to shorten the reads (e.g., nested PCR, sequence fragmentation) have to be carefully investigated before routine high-throughput sequencing. Consequently, the 18S and 28S rRNA genes are more suitable for investigating the phylogenetic relationship among higher rank fungal taxa, while the ITS region can be used alone or in combination with other protein-coding genes for genus- to species-level taxonomic identification [76]. Hence, it is important to recognize and account for biases and limitations inherent to universal barcodes, especially in fungal studies, where the primer selection might have a significant impact on the taxonomic identification.

#### 3.2.3. Identification of Protists from Environmental Samples

The major issue when selecting a primer pair for protists is the paraphyletic nature of this group. Protists are composed of all eukaryotic clades except Fungi, Metazoa and Embryophyta (i.e., higher plants). Except for a few protist clades that are found almost exclusively in marine environments (e.g., Diplonemea, Picozoa, Radiolaria, Telonemia, see [121]), all other clades were detected in soil, and thus only general eukaryotic primer can cover the complete biodiversity of terrestrial protists. Analogous to prokaryotes, the 18S rRNA gene has established as the standard gene for protist metabarcoding. The hypervariable regions V4 and V9 are the most commonly used, but multiple other hypervariable regions have been identified as suitable to cover the diversity of protists [122]. The EMP selected the primer pairs 1391F and EukBr targeting the V9 region for their standard protocol [123,124] while multiple other studies use slight variants with the primer 1380F/1389F and 1510R [125,126] (Table 5).

In parallel, the V4 region has also been established as an equally powerful region to resolve protist diversity when amplified with the TAReuk primer pair [132,136]. Other primer pairs have been designed to target V1-V3, V4-V5 and V7 regions, and they cover the biodiversity of protist clades well (see Table 5). However, no comparison has been thoroughly conducted of the performances of these primer pairs on terrestrial samples, and only in silico studies are available comparing them with the bias of database completeness for each region [18,121,122]. Moreover, considering that the Illumina sequencing of 2 × 300 bp now delivers almost identical quality to the 2 × 150 bp variant, a promising combination of primer amplifying 400 to 500 nt spanning regions can be tested like, for example, the V7 to V9 regions. The same primers have been used to study plant-associated protists. This is, for example, the case of *Sphagnum* and peatland-mosses-associated protists for which both V4 (TAReuk) and V9 (1380F/1510R) primers have been used [137,138]. Both V4 (V4_1f/TAReukREV3) and V9 (1380F/1510R) primers have also been employed to study rhizospheric protists [139,140]. Although plant sequences could represent the majority of reads in such plant-associated protist metabarcoding datasets, strategies to reduce the co-amplification of the associated plant(s), for example, the utilization of blocking oligos, have not yet been implemented. Furthermore, the use of general eukaryotic primers can come at the cost of reduced taxonomic coverage, which is not limited anymore by the primers and sequencing depth but by the competition between all target DNA during the PCR amplification. Indeed, specific primers have been shown to cover two to three times more diversity than general eukaryotic primers [141]. Likewise, clades often under-represented in general eukaryotic datasets, like Myxomycetes, can be recovered with clade-specific primers [142]. Lists of clade-specific primer pairs targeting either the same gene (18S) or other genes (e.g., 28S, ITS, COI, *rbc*L) are provided elsewhere [143,144].

### 3.3. Further Recommendations for Library Preparation

Once a proper primer pair has been selected, the library preparation workflow should be checked and evaluated for its compatibility with the chosen sequencing platform. In the case of Illumina sequencing technology, adaptor sequences and short barcodes must be added to the target gene primer sequence to enable the sequencing of many samples in parallel. This can be achieved with three different approaches. The Illumina standard workflow recommends a two-steps procedure in which the template is first amplified with the target gene primers that include the Illumina’s adaptors, while barcodes are added in a second PCR [68]. The second procedure involves only a single PCR step, in which the primers already incorporate the barcodes and adaptors [60]. This latter approach is used and recommended by the Earth Microbiome Project [61]. The third alternative is to perform the first PCR as for the Illumina standard workflow, and then to use a ligation-based kit, originally developed for shotgun sequencing, in order to reduce cost and avoid potential cross-contamination during the second PCR [145]. For this third approach, it is important to note that different steps in the ligation protocol (e.g., blunt ending, post-ligation PCR) can considerably increase the amount of tag-jump (sequencing outputs with false forward and reverse combinations of used tags) when pooling multiple tagged amplicons in the same library, and that adaptation of original kit protocol is necessary [146].

Several other factors related to library preparation and sequencing technology can significantly influence the accuracy of the metabarcoding procedure. For example, it is advisable to perform technical replicates for each sample during the PCR step and subsequently pool them before sequencing. This procedure allows one to minimize PCR-introduced biases on relative abundance and to efficiently saturate the diversity estimates of soil microbes [147]. To further reduce primer bias in the amplification process, it is important to determine the optimal annealing temperature for the primer pair chosen to avoid the formation of unspecific products. The optimal annealing temperature was found to be a function of the melting temperatures of the primers [148], and it should be determined empirically usually using the gradient PCR method. The use of proofreading DNA polymerases is strongly recommended to reduce chimera formation during PCR amplification, which may result in an overestimation of community richness [149].

Another important argument to consider is that Illumina sequencing platforms are known to causes biases when sequencing DNA libraries with low gene diversity, such as samples containing exclusively 16S rRNA gene or ITS amplicons [49,150]. To artificially increase sequence diversity, especially in the primer region, the addition of genomic DNA from the phage PhiX to the amplicon library is a common procedure. On the other hand, this results in a loss of sequence recovery because between 5 and 50% of the capacity of an Illumina sequencing run may have to be allocated to PhiX DNA sequencing. However, the amount of PhiX DNA to be used varies between Illumina platforms [151]. Alternatively, the design of heterogeneity spacers, short sequences of 1–7 bp linked to index adaptors or the gene-specific primers, can be utilized to reduce the amount of Phix DNA added to amplicon library pools to create the base diversity needed [152,153]. However, designing index adaptors or primers comprising different variable-length sequences can be a complicated and challenging approach with additional technical limitations [154]. However, this approach has been tested for multiple targets and allowed for an increased reads recovery and increased base quality at the 3′ end [155,156,157,158]. Another possibility to increase the base diversity is to sequence multiple targets in the same sequencing run (i.e., 16S, 18S and ITS gene libraries of the same samples), which is pertinent in research projects interested in multiple target taxa but should be restricted to marker gene of comparable length. 

The addition of negative controls is needed in order to estimate potential contamination during the DNA extraction and PCR preparation. It is thus recommended to use negative controls during each DNA extraction and each PCR preparation [8]. For DNA extraction, soil or plant material can be replaced with sterile water to create the negative control. This extracted material will then be used in PCR as a template to control for contamination during the DNA extraction. PCR negative controls use sterile water to replace DNA template in PCR in order to check for contamination during the PCR preparation. Even if no bands are visible on agarose gels for these negative controls, it is necessary to include them in the sequencing pool in order to detect potential low abundant contaminants. Sequences assigned to a PCR negative control need to be removed from any other sample from which the DNA was PCR-amplified together with this control. A particular case may arise when using double-tagging, as tag-jump could potentially produce sequences with an unused combination of tags by recombination of sequences from different samples in the sequencing pool. In such a situation, sequences assigned to negative controls by their tags could originate from other original samples and would thus contain a set of sequences mainly composed of the most abundant sequences found in the other samples sharing the same forward or reverse tag. Consequently, double tagging has to be used with caution, and multiple approaches have been developed to mitigate this issue [155,159].

The addition of mock communities (DNA pools of multiple known species) or positive controls (single-species DNA) into run libraries is also a common practice that can be helpful to (i) assess the primer bias and error rate of the sequenced run, (ii) benchmark bioinformatic tools, (iii) control for false positive in the case of tag-jumping, (iv) determine a relative abundance threshold to remove putative artifact out and (v) correct for compositional bias in case of differential abundance analyses. Initial Illumina MiSeq metabarcoding studies combining error rate estimates and bioinformatic tool benchmarking were based on sequencing bacterial, fungal and protists mock communities [135,160,161]. In general, mock communities are needed to validate new molecular (e.g., primer evaluation) and bioinformatic (e.g., sequence grouping algorithm) methods but are not crucial to analyze samples with established methodologies. Mock or positive controls can also be used to determine a threshold below which an OTU can be considered as an artifact. This threshold can be either a fixed number of reads [142] or a per-sample relative abundance when multiple positive controls were sequenced [162]. Most recent studies advocate for the use of separate or spike-in mock communities in order to use the recovered relative abundance of the known mixed species to apply a correction factor to a sample’s relative abundances [163]. This approach appears to be particularly crucial in differential abundance analyses when taking into account the compositional bias of amplicon sequencing data [164,165].

## 4. Bioinformatic Processing

### 4.1. Pre-Processing of the Metabarcoding Dataset

The typical metabarcoding bioinformatics pipeline consists of several steps, including (i) the demultiplexing of barcoded samples, (ii) pair-end assembly, (iii) removal of chimeric reads, (iv) quality filtering, (v) sequence grouping and (vi) comparison of the representative sequences to a reference database (Figure 1). QIIME and MOTHUR are the most-used platforms to perform bioinformatic analyses of metabarcoding data [166,167]. These software pipelines provide the capability to customize the analysis of high-throughput metabarcoding data using a wide choice of tools. However, many other pipelines and bioinformatics tools have been developed for the processing of amplicon sequencing data, such as PEMA [168], PipeCraft [169], SLIM [170], BioMas/Galaxy [171], PIPITS [172] USEARCH [173], VSEARCH [174], OBITools [175] and DADA2 [176]. Most of the above-mentioned platforms and pipelines are particularly well-suited for beginners in the field because they provide smooth wrappers around commonly used command-line tools as well as well-documented tutorials and examples [177]. It is important to note that some equivalent tools have been preferred in the analyses of certain target genes due to preference among the scientific communities, but most of them can be used for any metabarcoding target.

After the filtering and quality procedures, a key step in the bioinformatics analysis workflow is the clustering of reads based on their homology. Traditionally, during clustering, reads sharing a predefined level of similarity (generally between 95% and 99%) are assembled into Operational Taxonomic Units (OTUs) [178]. This step is intended to eliminate erroneous sequences produced by PCR and sequencing errors [18] as well as to merge intraspecific variance on diverging alleles or gene copies. However, such a global OTU clustering approach has several limitations [179]. For example, the 97% similarity cut-off used for V4 16S is to a large degree arbitrary, since different taxa might differ by a small percentage in their nucleotide sequence but still represent ecologically distinct clades [180,181]. In other words, there might be the risk that multiple similar species can be grouped into one single OTU with their true individual identifications being lost, while on the other hand, reads of a unique species may end up in different OTUs when the intra-specific variability is high. Other disadvantages of this method are associated with (i) the addition of data outputs, such as OTUs, that exclusively consist of PCR amplification or sequencing errors and (ii) the biologically meaningful interpretations/annotations of the inferred OTUs [181].

Recently, novel methods that use either single-linkage local clustering or error model correction algorithms have been developed to produce high-resolution representative sequences independently from a determined similarity threshold. The first approach was developed in the tool Swarm [182,183]. It allowed tackling the main issue of arbitrary similarity threshold of the global clustering approach. Swarm has allowed better discrimination of reads from closely related species, which is acknowledged by its wide adoption in the analysis of 18S rRNA metabarcoding datasets [136,184]. The second approach is called oligotyping [185] and is now mainly computed using the algorithm DADA2 [176]. DADA2 has been developed to control errors sufficiently to produce amplicon sequence variants (ASVs) that can be resolved exactly, down to the level of single-nucleotide differences over the sequenced region. This approach avoids clustering sequences at an arbitrarily defined similarity threshold (e.g., 97%) and instead uses only unique, identical sequences for downstream community analyses. Furthermore, because ASVs are exact sequences generated without clustering or reference databases, ASVs output can be readily compared between studies using the same target region and the same primers [186]. Several studies have reported that ASV-level pipelines allow for easier inter-study integration of biological features, as ASVs have intrinsic biological meaning, independent of reference database or study context [187,188,189]. The ASVs approach has also been described as being more effective than OTU clustering for recovering richness and composition of fungal [190] and bacterial [191] communities from environmental samples. Indeed, the DADA2 algorithm has shown to find more ASVs than other denoising pipelines when analyzing sequencing data from soil datasets, suggesting that it could be better at finding rare organisms, but at the expense of possible false positives [192]. For the aforementioned reasons, most of the recent metabarcoding studies on bacterial and fungal microbiota associated with soil and plant material have chosen ASVs over OTUs [193,194,195,196,197,198]. However, fungal and bacterial diversity patterns appear to be equally well described by both OTU and ASV, which does not appear to change the conclusion on alpha and beta diversity analyses over contrasted samples along elevation gradients [199].

For a meaningful interpretation and reliable analysis of amplicon sequencing data, after the OTU/ASVs generation stage, additional steps should be considered. Primarily, post-clustering algorithm should be used when a high amount of artefactual sequence variants are suspected [200,201]. Then, an adequate coverage, in terms of sequencing depth, is crucial to generate reliable information on the composition and taxonomic structure of the microbial community investigated. Rarefaction and accumulation curves can provide useful information to assess whether the sequencing depth yielded sufficient reads to describe most of the diversity in the samples. For example, if the coverage per sample is too low, the diversity of the microbiota being studied is likely to be underrepresented, as rarer members of the microbiota are less likely to be detected [19]. In general, a satisfactory coverage can be achieved with 10,000 to 100,000 sequences per sample, but it largely depends on the complexity of the microbiota, type of starting material (soil or plant), the targeted gene and the desired resolution [35].

Additional filtering steps will increase the quality and resolution of the output dataset. For example, the exclusion of rare OTUs or ASVs, which may be sequencing artifacts, is commonly recommended [202]. However, there is no consensus on the threshold number of sequences below which an OTU/ASV can be considered rare [190]. The suggested thresholds might range from 1 to 10 sequences [203] or depend on the relative abundance of OTUs/ASVs [160]. Another filtering option is to remove OTUs/ASVs that have been detected solely in one or a few samples from a single sequencing run, but such an approach strictly depends on the number of samples that constitute the entire dataset and if multiple sequencing runs were used. 

### 4.2. Taxonomic Profiling

The taxonomic annotation of the OTUs/ASVs identified is the last step of the metabarcoding workflow. It provides valuable information on the OTUs/ASVs in light of what is known about these taxa from previous works, and, more broadly, it allows comparison across microbiota studies [18]. Essentially, the taxonomical identification of microbes relies on sequence similarity searches in reference databases. It is noteworthy that taxonomy assignment based on different reference databases might lead to different results [204]. So far, there is no consensus on which reference database to use for taxonomic assignment of the detected OTUs/ASVs. In this section, we report the most common options utilized by bioinformaticians and microbial ecologists.

Reference databases for 16S rRNA gene taxonomy assignment include SILVA [205], the Ribosomal Database Project (RDP) [206], Greengenes [207] and the National Center for Biotechnology Information (NCBI) [208]. Since all these databases are widely used for taxonomical identification of prokaryotic sequences, we provide here a quick overview of each of them (Table 6). 

The SILVA database provides a phylogenetic classification for the small and large rRNA subunits for Bacteria, Archaea and Eukarya in the European Nucleotide Archive (ENA) [211]. It is based primarily on phylogenies for small subunit rRNAs (16S rRNA gene for prokaryotes and 18S rRNA gene for eukaryotes), and its taxonomic rank assignment is manually curated. To date, the last SILVA database update was on 27.08.2020 with the 138.1 release. Interestingly, the QIIME2 platform makes available pre-formatted SILVA reference databases to QIIME2 users in order to provide a fast and standardized workflow in the taxonomy assignation step. The RDP database also contains rRNA sequences from the three domains, but it provides primarily phylogenetic classification for prokaryotic organisms. It contains sequences available from the International Nucleotide Sequence Database Collaboration (INSDC) [212]. The RDP classifier was updated to version 2.13, which was released on 30 July 2020. Greengenes is a database that provides a phylogenetic classification of prokaryotic organisms, and most of the sequences are retrieved from the NCBI GenBank [213]. The last update of the Greengenes database occurred on 5 January 2019. The NCBI taxonomy database contains the names of all organisms associated with submissions to the NCBI sequence databases. Specifically, the NCBI Taxonomy database is the standard nomenclature and classification repository for the International Nucleotide Sequence Database Collaboration (INSDC), comprising the GenBank, European Nucleotide Archive (ENA) and DNA Data Bank of Japan (DDBJ) databases [208].

For the taxonomic identification of fungi, three main reference ITS databases spanning the fungal kingdom are available: UNITE [209], Warcup ITS [214] and RDP. Among them, UNITE is considered as the main reference ITS database for the identification of fungi. It represents a middle ground between including the very latest sequences and offering detailed taxonomic annotation [95]. Indeed, UNITE clusters the ITS sequences at different sequence similarity thresholds to obtain approximate species-level OTUs referred to as species hypotheses (SHs) [215]. These SHs (458,797 as of August 2018) have a unique digital object identifier (DOI) to allow stable, unambiguous reference across studies [216]. Its last update was on 20 February 2020 with the release version 8.2. It is worth noting the existence of two ITS reference databases sequences associated with a specific ITS sublocus. This is the case of ITSoneDB [217], which is a curated collection of eukaryotic ITS1 sequences, and the ITS2 Database [218], which is a eukaryotic ITS2 database.

Other reference databases for fungal annotation are used if the target marker gene amplified via PCR differs from the ITS region, such as LSU or SSU regions of the fungal rRNA gene. In this case, SILVA, RDP and NCBI databases are ubiquitously employed. Interestingly, for the specific taxonomic classification of fungal taxa affiliated to the phylum Glomeromycota, the MaarjAM database [219] was created in 2010. This database associates information about geography, habitat and climate to Glomeromycota sequences, which cluster in “Virtual Taxa”, a proxy for fungal species [220]. The MaarjAM database is manually curated, and its last update occurred on 5 June 2019.

The main reference database for the eukaryotic 18S rRNA gene is the Protist Reference Database (PR2; [210], now accessible at https://github.com/pr2database/pr2database, accessed on 13 January 2021). It is a curated reference 18S sequence collection that follows the most up-to-date higher ranks taxonomic classification of eukaryotes [143]. The classification is provided in a fixed eight-rank taxonomy, which eases the statistical analyses. The last version is 4.12.0 from 8 August 2019. Alternatively, the SSU Ref NR 99 SILVA reference database can also be used, which can be particularly interesting when using the aligned version of the database.

Overall, the selection and availability of curated reference databases are crucial to characterize on a large scale the taxonomic complexity of microbiota from various environments through metabarcoding.

## 5. Importance of Metadata Standards and Archiving Practices

As DNA metabarcoding has become a routine approach for the characterization of microbial communities across different environments, in recent years a surge in the volume of the sequences archived in public genetic repositories has been recorded [221]. Presently, the deposition of sequencing data in genetic databases has become standard practice, mainly because it is a more frequent requirement for the publication of studies in peer-reviewed journals. The electronic archiving of sequencing data is primarily centralized in three public genetic databases that are routinely synchronized and members of the INSDC: NCBI’s Sequence Read Archive (SRA), the EBI’s European Nucleotide Archive (ENA) and DDJ’s Sequence Read Archives (DRA) [212]. These archives represent an invaluable resource as they create a window of opportunity for data reuse and synthesis in microbiome research. Therefore, it is crucial that the sequencing data are correctly uploaded and made available in public genetic repositories with appropriate formatting and metadata to allow others to reuse them. The standardization of protocols and metadata collection, alongside a simple and straightforward process of data storage, accessibility and sharing, is vital for ensuring that microbiome data are findable, accessible, interoperable and reusable (FAIR) [222].

Several research groups and consortiums have pioneered and coordinated the generation of community-driven standards for collecting and managing relevant contextual information associated with genomic data. So far, the minimum information standards (MIxS: minimum information about any (x) sequence) established by the Genomic Standards Consortium (GSC) [223] is the most accepted and adopted initiative by the public genetic databases in order to provide rich information on the uploaded sequences [224]. The MIxS standards consist of checklists for describing minimum information about marker genes (MIMARKS), genomes (MIGS) and metagenomes (MIMS), and of 15 different environmental packages that can be used to specify the environmental context of a sequenced microbial community, particularly for soil and plant-associated samples [225]. In parallel, MIMARKS standards have been developed by GSC for reporting information about metabarcoding studies [226], and the MIMARKS checklist is provided on the GSC website (https://gensc.org/mixs/, accessed on 13 January 2021). The implementation of this checklist alongside the sequencing data is fundamental to facilitate the ability to retrieve appropriate contextual information for marker genes, frequently referred to as “metadata”, enabling the reusability and sharing of the sequencing data to allow for reproducibility, meta-analyses and cross-comparison among studies.

Although many efforts have been made to demonstrate and promote the importance of having systematic reporting conventions and standards to accurately describe any chosen workflow, a recent study on the deposited sequencing data of 26,927 microbial studies published between January 2015 and March 2019 showed gaps in the availability and reusability of these data [227]. The authors of this study identified the lack of metadata, improper file formatting and data deposition to inappropriate repositories as the main causes of data loss. In particular, the lack or the incorrect information reported in the metadata, which includes all information concerning the description of the sample, sample processing, experimental design, library creation and sequencing platform configuration, represents a common issue that hinders the reusability of the sequencing data available in genetic databases. In light of these findings, we would like to emphasize the importance of improving data archiving practices to enhance the value of the sequencing data in repurposing and better sharing of microbial datasets.

## 6. Future Perspective and Challenges

Within the past decade, metabarcoding has become the gold standard for the characterization of complex microbial communities associated with environmental samples. Although this approach may not successfully identify all the taxa in a sample, the output generated by a proper metabarcoding workflow provides reliable information for adequate biological inferences. However, generating accurate and verifiable data, such as biodiversity estimates and taxonomic assignation, requires robust methods and generally accepted standards [228]. So far, metabarcoding workflows have relied primarily on Illumina sequencing technology, which constrains the length of the amplicons to a maximum of 600 bp. This represents a considerable limitation in terms of taxonomic resolution for many bacterial and fungal taxa, as the taxonomic assignment of short-reads at the species or even genus level is often elusive. Third-generation sequencing technologies, such as the MinION and PromethION platform from Oxford Nanopore Technologies (ONT) or PacBio from Pacific Biosciences, are emerging as promising sequencing systems to overcome many of the limitations of short-read sequencing. Considering that ONT technology allows for the design of primers covering the whole length of the 16S rRNA gene or ITS region, it is then plausible to conceive a better phylogenetic inference and higher taxonomic resolution in microbial ecology studies. However, despite the apparent potential advantages of the application of ONT technology in metabarcoding, there are still several factors limiting its implementation in microbial ecology research. For instance, there is only a limited number of bioinformatic tools and protocols designed for the specific analysis of long reads. Thus, it is challenging to carry out a specialized taxonomic analysis compared with previous sequencing technologies [229]. Another major drawback of this technology is the high read error rates, which hampers accurate read classification [230]. Furthermore, it is a relatively novel technology for which standards are still largely absent, thus complicating the standardization and reproducibility of results [231].

Other methodological approaches can also be employed in the characterization of complex microbiota from environmental samples. Metagenomics, or the shotgun sequencing technique, which refers to the recovery and sequencing of the collective genomic material in environmental samples, are largely used to investigate the functional complement of the microbiota as a whole. Nonetheless, the data output generated by this approach can also be utilized for taxonomic profiling. A significant advantage of metagenomics over metabarcoding is that metagenomic approaches do not rely on the amplification of specific genomic sequences, avoiding all the bias introduced by PCR procedures. However, important drawbacks are associated with shotgun sequencing in biodiversity studies. The efficiency of shotgun metagenomics is mainly constrained to adequate read depths in order to obtain accurate results, which can be difficult to achieve from complex samples like soil. Hence, huge increases in sequencing power to acquire adequate sequencing depth often result in prohibitive costs. Another main disadvantage associated with shotgun metagenomics is the lack of curated reference databases of bacterial and fungal genomes. Specifically, fungal and protist genome databases are rare at present and, in particular, compared with bacterial genome databases [95,232]. As a result, the proportion of sequences identified as fungal is low even in metagenomes with high fungal abundance, such as topsoil metagenomes [233]. Lastly, challenges and difficulties frequently occur in analyzing metagenomics datasets because of the extensive filtering that is required as a result of the sequencing of all sampled DNA. This leads to datasets of significantly larger orders of magnitude compared to the ones produced by metabarcoding approaches. Consequently, analyses of shotgun metagenomics data take much longer to perform and require far more computational power and expertise. 

Capture by hybridization also represents a promising approach for the enrichment of a target gene as an alternative to PCR amplification [234]. It has the advantage of allowing the use of multiple probes annealing to the target gene and allows the conservation of long DNA fragments, which is suitable for third-generation high-throughput sequencing. This novel technique also has the potential to unravel new hidden diversity missed by the traditional PCR approach [235].

In conclusion, DNA metabarcoding represents a powerful approach to explore the microbial biodiversity of environmental samples. With further technological advances, procedure optimization and refinement, metabarcoding will likely emerge as a fundamental tool for several scientific tasks not only in biodiversity monitoring in terrestrial environments but also in other research and application areas such as diet analysis, air, water and food quality testing and monitoring [15]. Moreover, the future of DNA metabarcoding deeply relies on the quality and completeness of reference sequence databases, which should be also designed and further curated to allow efficient data mining and report generation. Finally, we believe that the combination of different sequencing methodologies, such as DNA metabarcoding and metagenomics, together with gene expression, including metatranscriptomics, stable isotope labeling and canonical cultivation and enrichment techniques, represents the best approach to open the soil black box in order to unravel the complex dynamics of the soil–plant–microbe system and to get further insight into soil microbial functions on the level of complex terrestrial microbiota.

## Figures and Tables

**Table 2:** Primer pairs targeting the 16S rRNA gene that have been frequently used to characterize Bacteria biodiversity in studies based on Illumina sequencing.

Primer Pair	Sequence 5′-3′	Tm (°C) *	Amplified Region	Amplicon Length	Reference
515fB	GTGYCAGCMGCCGCGGTAA	63.6	V4	253	[50]
806rB	GGACTACNVGGGTWTCTAAT	51.2			[51]
515fB	GTGYCAGCMGCCGCGGTAA	63.6	V4-V5	394	[50]
926r	CCGYCAATTYMTTTRAGTTT	48.9			[52]
341f	CCTACGGGAGGCAGCAG	58.2	V3-V4	418	[37]
B805r	GACTACHVGGGTATCTAATCC	51.3			
799f	AACMGGATTAGATACCCKG	50.9	V5–V6	301	[53]
1115r	AGGGTTGCGCTCGTTG	56.1			[54]
799f	AACMGGATTAGATACCCKG	50.9	V5-V7	377	[53]
1193r	ACGTCATCCCCACCTTCC	57.1			[55]
967f	CAACGCGAAGAACCTTACC	53.8	V6-V8	405	[56]
1391r	GACGGGCGGTGWGTRCA	59.5			[57]
68f	TNANACATGCAAGTCGRRCG	55.5	V1-V3	438	[58]
518r	WTTACCGCGGCTGCTG	56			[59]

* Average melting temperature as calculated with OligoAnalyzer using default parameter (www.idtdna.com/calc/analyzer, accessed on 13 January 2021).

**Table 3:** Primer pairs targeting the 16S rRNA gene that have been frequently used to characterize Archaea biodiversity in studies based on Illumina sequencing.

Primer Pair	Sequence 5′-3′	Tm (°C) *	Amplified Region	Amplicon Length	Reference
515fB	GTGYCAGCMGCCGCGGTAA	63.6	V4	253	[50]
806rB	GGACTACNVGGGTWTCTAAT	51.2			[51]
340f	CCCTAYGGGGYGCASCAG	61.3	V3-V4	388	[63]
806rB	GGACTACNVGGGTWTCTAAT	51.2			[51]
SSU1ArF	TCCGGTTGATCCYGCBRG	59.2	V1-V4	491	[62]
SSU520R	GCTACGRRYGYTTTARRC	51			
349f	GYGCASCAGKCGMGAAW	57.7	V3-V4	111	[64]
519r	TTACCGCGGCKGCTG	57.6			[37]
Parch519f	CAGCCGCCGCGGTAA	59.4	V4-V5	386	[65]
Arch915r	GTGCTCCCCCGCCAATTCCT	62.9			[66]
1106F	TTWAGTCAGGCAACGAGC	52.5	V7-V8	280	[67]
1378R	TGTGCAAGGAGCAGGGAC	57.9			

* Average melting temperature as calculated with OligoAnalyzer using default parameter (www.idtdna.com/calc/analyzer, accessed on 13 January 2021).

**Table 4:** Primer pairs targeting the ITS region that have been frequently used to characterize fungal biodiversity in studies based on Illumina sequencing.

Primer Pair	Sequence 5′-3′	Tm (°C) *	Amplified Region	Amplicon Length	Reference
ITS1f	CTTGGTCATTTAGAGGAAGTAA	49.7	ITS1	357	[99]
ITS2r	GCTGCGTTCTTCATCGATGC	57			
ITS1F_KYO2	TAGAGGAAGTAAAAGTCGTAA	48	ITS1	358	[100]
ITS2_KYO2	TTYRCTRCGTTCTTCATC	48.4			
ITS3	GCATCGATGAAGAACGCAGC	57	ITS2	306	[99]
ITS4	TCCTCCGCTTATTGATATGC	52.1			
gITS7	GTGARTCATCGARTCTTTG	48.3	ITS2	288	[101]
ITS4ngs	TTCCTSCGCTTATTGATATGC	52.9			[102]
fITS7	GTGARTCATCGAATCTTTG	47.3	ITS2	292	[101]
ITS4	TCCTCCGCTTATTGATATGC	52.1			[99]
ITS86f	GTGAATCATCGAATCTTTGAA	48.6	ITS2	290	[103]
ITS4	TCCTCCGCTTATTGATATGC	52.1			[99]

* Average melting temperature as calculated with OligoAnalyzer using default parameter (www.idtdna.com/calc/analyzer, accessed on 13 January 2021).

**Table 5:** Primer pairs targeting the 18S rRNA gene that have been frequently used to characterize protists biodiversity in studies based on Illumina sequencing.

Primer Pair	Sequence 5′-3′	Tm (°C) *	Amplified Region	Amplicon Length	Reference
NS1/Euk20f	GTAGTCATATGCTTGTCTC	47.2	V1-V3	507	[99,127]
Euk516r	ACCAGACTTGCCCTCC	54.3			[128]
18S_0067a_deg	AAGCCATGCATGYCTAAGTATMA	54.4	V1-V3	310	[129]
NSR 399	TCTCAGGCTCCYTCTCCGG	59.7			
fw_366	ATTAGGGTTCGATTCCGGAGAGG	58.2	V3	180	[130]
rv_586	CTGGAATTACCGCGGSTGCTG	61			
TAReuk454FWD1/V4_1f	CCAGCASCYGCGGTAATTCC/CCAGCASCYGCGGTAATWCC	60.1/59.9	V4	391	[131]
TAReukREV3	ACTTTCGTTCTTGATYRA	45.9			[132]
616*f	TTAAARVGYTCGTAGTYG	47.1	V4-V5	504	[133]
1132r	CCGTCAATTHCTTYAART	45.4			
18S_allshorts-f	TTTGTCTGSTTAATTSCG	47.7	V7	109	[134]
18S_allshort-r	TCACAGACCTGTTATTGC	49.4			
V8f	ATAACAGGTCTGTGATGCCCT	55.9	V8-V9	339	[135]
1510R	CCTTCYGCAGGTTCACCTAC	56.6			[125]
1380F/1389F	CCCTGCCHTTTGTACACAC/TTGTACACACCGCCC	54.6/51.9	V9	141/136	[125]
1510R	CCTTCYGCAGGTTCACCTAC	56.6			
1391F	GTACACACCGCCCGTC	56.1	V9	127	[123]
EukBr	TGATCCTTCTGCAGGTTCACCTAC	58.4			[124]

* Average melting temperature as calculated with OligoAnalyzer using default parameter (www.idtdna.com/calc/analyzer, accessed on 13 January 2021).

**Table 6:** List of the main reference databases used for the taxonomic annotation of the representative sequences in metabarcoding studies of terrestrial microbial communities.

Database/Release	Marker/Taxa	URL *	Reference
SILVA/138.1	16S, 18S SSU, 23S, 28S, LSU rRNA sequences/Archaea, Prokaryotes, Eukaryotes	www.arb-silva.de	[205]
Ribosomal Database Project (RDP)/11	16S, 28S rRNA sequences/Prokaryotes, Archaea and Fungi	rdp.cme.msu.edu	[207]
Greengenes/12_10	16S rRNA sequences/Archaea and Bacteria	greengenes.secondgenome.com	[207]
National Center for Biotechnology Information (NCBI) GenBank/241.0	raw sequences/Archaea, Prokaryotes, Eukaryotes	www.ncbi.nlm.nih.gov	[208]
UNITE/8.2	nuclear ribosomal ITS region sequences/Eukaryotes	unite.ut.ee	[209],
Protist Reference Database (PR2)/4.12.0	18S rRNA sequences/Eukaryotes	pr2-database.org	[210]

*, accessed on 13 January 2021.
